# Spatial and Temporal Distribution of Pediatric Cancers in Southern Ghana: A Retrospective Observational Study

**DOI:** 10.1002/hsr2.70797

**Published:** 2025-04-29

**Authors:** Efiba Vidda Senkyire Kwarteng, Amma Aboagyewa Larbi, Nanatte Veronica Anderson, Sheriff Wahab, Yaa Gyamfua Oppong‐Mensah, Samuel Ato Andam‐Akorful, Alexander Kwarteng, Lawrence Osei‐Tutu, Gladys Acquah, Comfort Asoogo, Bernice Eklu, Paul Obeng, Barnabas Manlokiya, Ben Gyan, Vivian Paintsil

**Affiliations:** ^1^ Department of Geomatic Engineering Kwame Nkrumah University of Science and Technology Kumasi Ghana; ^2^ Department of Biochemistry and Biotechnology Kwame Nkrumah University of Science and Technology Kumasi Ghana; ^3^ Department of Geology and Geological Engineering University of Mississippi Mississippi USA; ^4^ Directorate of Child Health Komfo Anokye Teaching Hospital Kumasi Ghana; ^5^ Kumasi Centre for Collaborative Research in Tropical Medicine Kwame Nkrumah University of Science and Technology Kumasi Ghana; ^6^ Noguchi Memorial Institute for Medical Research University of Ghana Ghana; ^7^ Department of Child Health School of Medical Sciences, Kwame Nkrumah University of Science and Technology Kumasi Ghana

**Keywords:** leukemias, lymphomas, nephroblastoma, pediatric cancer, spatial distribution

## Abstract

**Background:**

More than 80% of children living with cancer reside in developing countries like Ghana, with over 1000 new cases projected each year. Spatial analysis of pediatric cancers provides insights into possible environmental risk factors, potential genetic associations, and the identification of clusters or hotspots, which can guide further investigation into causal factors and inform population‐level interventions.

**Aim:**

This retrospective observational study aimed to investigate the spatial distribution dynamics of pediatric cancer cases diagnosed at the Komfo Anokye Teaching Hospital, Ghana.

**Methods:**

Poisson regression and spatial statistical tools, including Kernel Density Estimation (KDE) and Kulldorff's spatial scan statistics, were used to analyze 652 pediatric cancer cases recorded from 2018 to 2022 in Southern Ghana.

**Results:**

The results revealed increasing pediatric cancer cases over the 5 years. Burkitt Lymphoma, Leukemia, Nephroblastoma, Other Non‐Hodgkins Lymphoma, and Retinoblastoma had higher incidence among children, with males having a 24% higher incidence rate compared to females (Incident Rate Ratios [IRR] estimated at 1.24 at *p* < 0.01, 95% confidence interval [CI): 1.06, 1.45). Further, the KDE plot consistently revealed a high density of reported cases in the central part of the study area, with a noticeable directional diffusion of pediatric cancer cases toward the northwest from 2018 to 2022. Three significant clusters of relative risk between 1.65 and 2.17, *p* < 0.01, were identified, covering parts of the Bono East and Ashanti Regions of Ghana.

**Conclusion:**

Clustering cancer cases could suggest a possible environmental influence on the occurrence of the disease. Although this study offers relevant baseline information, comprehensive epidemiological investigations are necessary to establish specific environmental risk factors and potential gene‐environment interactions contributing to this pediatric cancer clustering.

## 1 Introduction

For many years, cancer has persistently impacted global health and economy due to its high prevalence and mortality rates. It claimed 9.7 million lives and affected 20 million people worldwide in 2022 [1]. Although pediatric, adolescent, and young adult cancers are rare compared to adult cancers, the devastating effect it has on families and communities cannot be overstated. Pediatric cancers include but are not limited to leukemia, lymphoma, brain and spinal cord tumors, bone sarcomas, neuroblastoma, liver tumors, and germ cell tumors [2]. It is an undisputed fact that there are more older people than younger people (children and adolescents) living with cancer [3]. However, the relative rarity of pediatric cancer does not diminish the devastating impact it has on families and communities who suffer the loss of a young life to the disease.

The rate of pediatric cancer is currently 56.3 cases per million. It has been projected that Africa will account for half of all childhood cancer cases worldwide by 2050 [4]. According to currently available information, there are 124 and 103 new child cancer cases annually at Korle‐Bu Teaching Hospital and Komfo Anokye Teaching Hospital, Ghana's leading referral public institutions [5]. Recent unpublished data shows an increase of 140 new childhood cancer cases annually at Komfo Anokye Teaching Hospital. There is still a gap in diagnosis as the annual childhood cancer cases in Ghana are expected to be around 1,200 among children under the age of 15 [6]. The etiology of childhood cancers is largely unknown. However, age, sex, and race/ethnicity have long been linked to differences in the incidence of childhood cancer from different regions. The association between sex and the different childhood cancers is poorly understood.

Interestingly, males have a higher childhood cancer incidence than females in most of the major cancers. The overall male‐to‐female incidence rate ratio is estimated to be 1.19 (95% confidence interval [CI]: 1.18‐1.20), with specific cancers exhibiting ratios ranging from 1.13 (astrocytoma and neuroblastoma) to 4.62 (Burkitt lymphoma) [7]. In contrast, females are more susceptible to nephroblastoma, extracranial and extragonadal germ cell tumors, and thyroid carcinoma. The underlying biological mechanisms driving the association between male sex and increased childhood cancer risk remain elusive [8, 9]. Still, there is little information on other risk factors besides high‐dose radiation, pesticide exposure, and past treatment [10]. Geographical variations in childhood cancer incidence rates point to genetic and environmental impacts on disease susceptibility. Malnutrition, viral infections, social deprivation, and exposure to malaria are believed to have a significant effect on the pathology and clinical features of cancer [11]. Other environmental factors like humidity, pesticide use, air and water pollution, tobacco smoke, and exposure to carcinogens can contribute to the development of cancers, depending on the amount and duration of exposure and the individual's genetic background [12, 13].

In contrast to adult cancer, if a child is diagnosed with cancer and is given access to treatment and comprehensive care, 8 out of 10 of them will survive the condition, according to [14]. However, the opposite has proven true in resource‐poor nations like Ghana: barely 20% of children with cancer survive [6]. In Ghana, although there is an increased prevalence of pediatric cancers, several critical factors have contributed to the low survival. Amongst them are financial constraints faced by families, limited availability of quality childhood cancer drugs, insufficient incentives or motivation for healthcare workers, and lack of awareness among parents and guardians [15, 16, 17].

Thus, this study sought to identify spatial and temporal patterns of pediatric cancer in Southern Ghana and to identify areas of high incidence. Focusing on southern Ghana is crucial due to the region's disproportionately high pediatric cancer burden, exacerbated by rapid urbanization and environmental factors. Its strategic location and specialized services provide vital access for patients, making it an ideal site for research and intervention development to improve outcomes for children with cancer. The results provide valuable insights into the spatial distribution of pediatric cancer in Ghana and help inform policies aimed at identifying risk factors and mitigating impacts.

## 2 Methods

### 2.1 Study Design

This study utilized a retrospective observational study design [18]. Secondary data on pediatric cancer cases diagnosed at the Komfo Anokye Teaching Hospital (KATH) from 2018 to 2022 was used in this analysis.

### 2.2 Study Site

**Figure 1:**
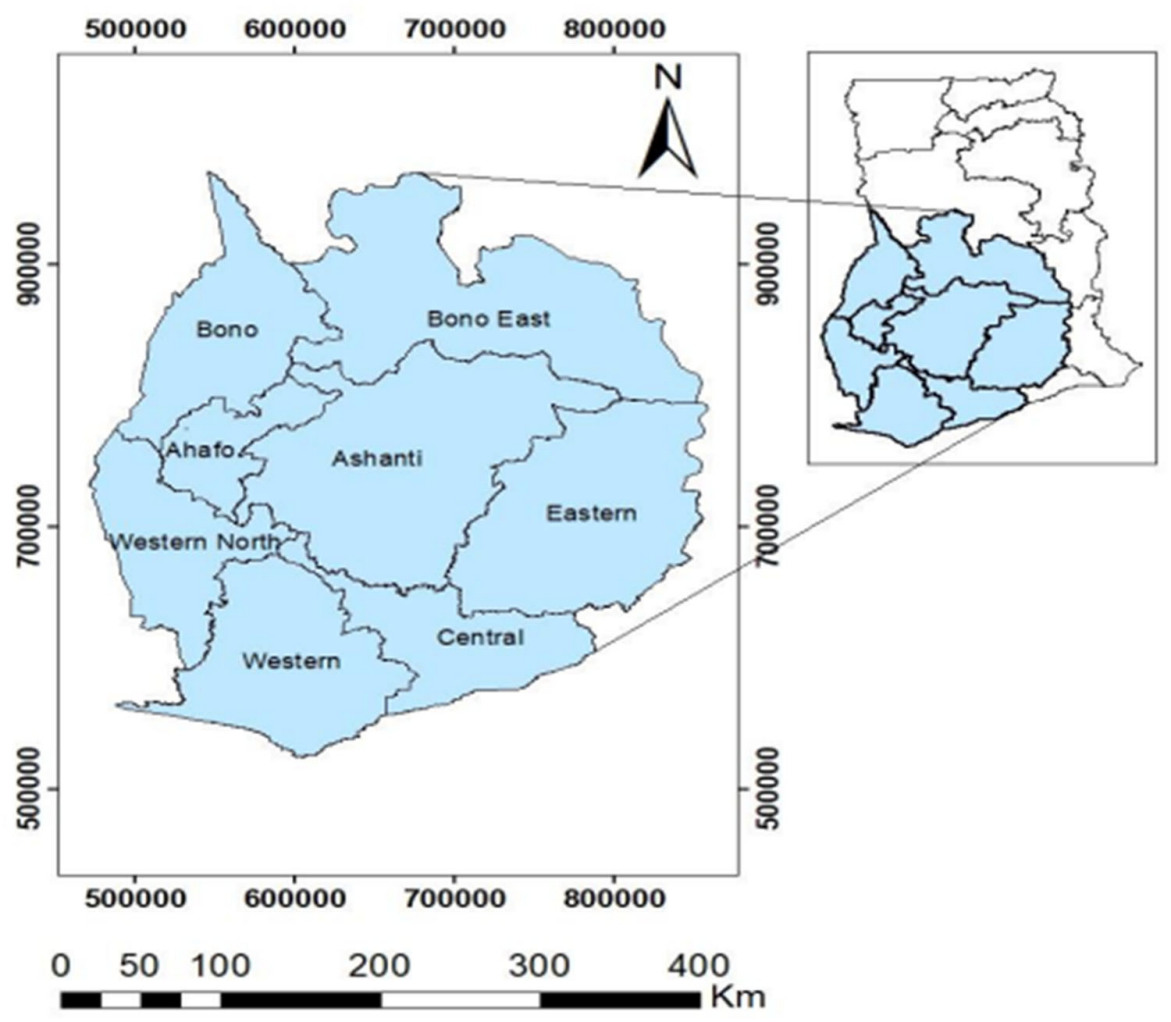
A map of southern Ghana (the study area), with the Ghana map as an insert map.

Ghana is in West Africa at 8°00'N and 2°00'W. From its southernmost to northernmost points, the nation stretches for roughly 540 km (335 miles); from its westernmost to easternmost points, it does so for around 280 km (174 miles). To ensure sufficient data for meaningful pattern detection while considering regions with higher concentrations of reported cancer cases, a 150 km buffer around KATH was overlaid on the Ghana map. Eight (8) regions within southern Ghana that fell within the buffer were selected for spatial analysis, as indicated in Figure 1. This approach enhances the reliability of spatial clustering results.

KATH is situated at the central business center of Kumasi in the Ashanti Region. It is a 1200‐bed hospital with various Clinical specialties. It is the home of the Kumasi Population‐Based Cancer Registry. The Pediatric Oncology unit is one of the subspecialties at the Directorate of Child Health and takes referrals from 12 out of the 16 regions of Ghana. Patients with all the different types of childhood cancer are seen and enrolled in the registry.

### 2.3 Data Collection and Preparation

The secondary pediatric cancer data was obtained from the Pediatric Oncology Unit at the Komfo Anokye Teaching Hospital. The 5‐year (2018–2022) data contained reported pediatric cancer cases, age, sex, diagnosis, and location of patients. To ensure patient confidentiality, patient addresses were aggregated to the community level resolution before geocoding, and all personally identifiable information was removed. The data was validated and verified by manually loading it into Google Earth Pro Version 10.76.0.1 for geographic verification (https://earth.google.com [19]). The data was stored in a Comma‐separated values (CSV) format and further analyzed in the Rstudio version 4.3.2 environment [20].

### 2.4 Gender‐Based Diagnosis and Temporary Pediatric Cancer Trend

To study the trend in cancer cases, Poisson regression modeling was used to assess the association between cancer incidence counts and the year of diagnosis while adjusting for cancer type and gender. Given that the outcome variable (count of cancer cases) follows a count‐based distribution, we assumed a Poisson process and fitted a generalized linear model (GLM) with a log‐link function as $(E\left(Y_{i}\right))=\;\beta_{0}+\beta_{1}\left(\mathrm{Year}\right)+\sum_{j=2}^{J}{\beta_{j}(\mathrm{Cancer Type}}_{i})+\beta_{k}{(\mathrm{Gender}}_{k})$, where $E\left(Y_{i}\right)\;$represents the expected number of cancer cases in Year i based on 5 years of data collected from 2018 to 2022. Cancer Type is a categorical predictor with multiple levels representing different cancer diagnoses and Gender was included as a binary variable (Male = 1, Female = 0 as the reference category). Since the Poisson regression model applies a log‐link function, the estimated coefficients (log‐scale estimates) were exponentiated to obtain incidence rate ratios (IRRs), $\mathrm{Rate Ratio}=\;e^{\mathrm{Estimates}}$, which represent the multiplicative effect of each predictor on cancer incidence. Model dispersion was assessed using the overdispersion test to check if the mean and variance of the count of cancer cases per category are equal i.e., E(Y)=Var(Y).

### 2.5 Density Analysis

To estimate the density of pediatric cancer cases within the defined study area, the kernel density estimation (KDE) technique was used [21]. KDE calculates the density of points by creating a smooth, continuous density surface representing the intensity of point occurrences. The KDE bandwidth was selected using the adaptive bandwidth method implemented in the ks package in R (RStudio version 4.3.2). This method dynamically adjusts the bandwidth based on local point density, assigning smaller bandwidths in high‐density regions and larger bandwidths in sparse regions to better capture spatial variations, reducing the risk of over‐smoothing or excessive noise. Additionally, we ensured robustness by visually inspecting the KDE output and comparing it with alternative bandwidth choices during the initial analysis phase. The temporal evolution of the disease was studied by performing KDE for each year.

### 2.6 Cluster Analysis of Pediatric Cancer

To provide insights into the spatial patterns and potential hotspots within the study area, Kulldorff's spatial scan statistic was utilized to detect the specific locations and extent of pediatric cancer (PC) clusters [22] in SaTScan v10.2.5. This method provides valuable information on cluster location, size, and temporal behavior, which is crucial for targeted intervention and monitoring. Additionally, identifying hotspot areas can guide decisions on where to site cancer care facilities, improving treatment and care.

The study employed several statistical methods to analyze the pediatric cancer data. Poisson regression modeling was utilized to examine the relationship between cancer incidence counts and the year of diagnosis, with adjustments for cancer type and gender. KDE was applied to estimate the spatial density of pediatric cancer cases within the study area. To identify potential spatial clusters and hotspots, Kulldorff's spatial scan statistic was implemented. All analyses were conducted with R version 4.3.2 and SaTScan v10.2.5.

## 3 Results

**Figure 2:**
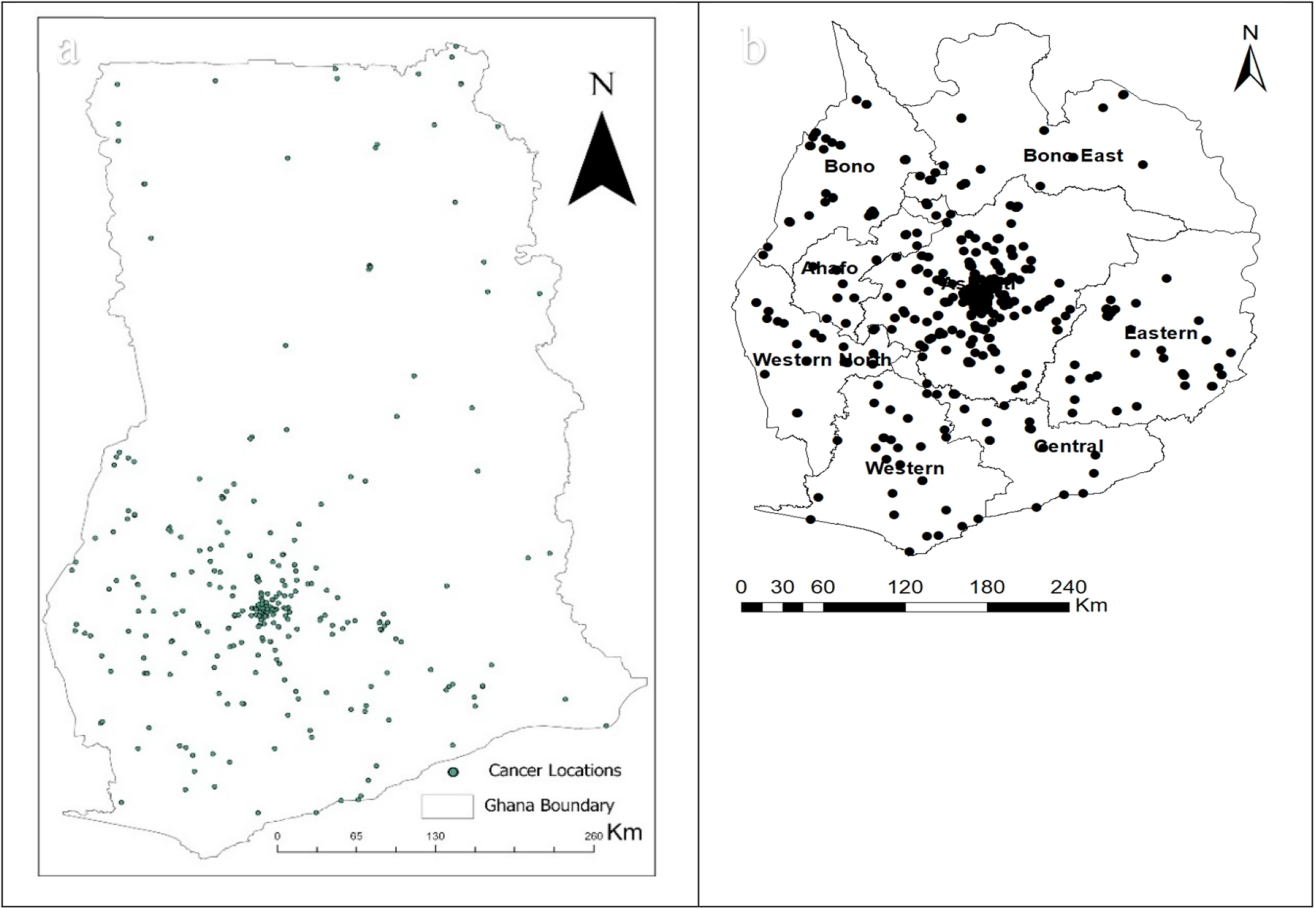
A map of all cancer cases recorded at KATH from 2018 to 2022. (a) All cases across the country, and (b) cases in the South.

This study conducted a comprehensive spatial analysis of cancer incidences in southern Ghana. Overall, a total of 652 pediatric cancer cases were recorded over the 5 years, from 2018 to 2022 Figure 2. The distribution shows that most cases were reported from southern Ghana, with a notable concentration in the Ashanti region (Figure 2a,b). Very few cases were recorded from the northern part of the country. This could be attributed to KATH's accessibility as a major referral center.

**Figure 3:**
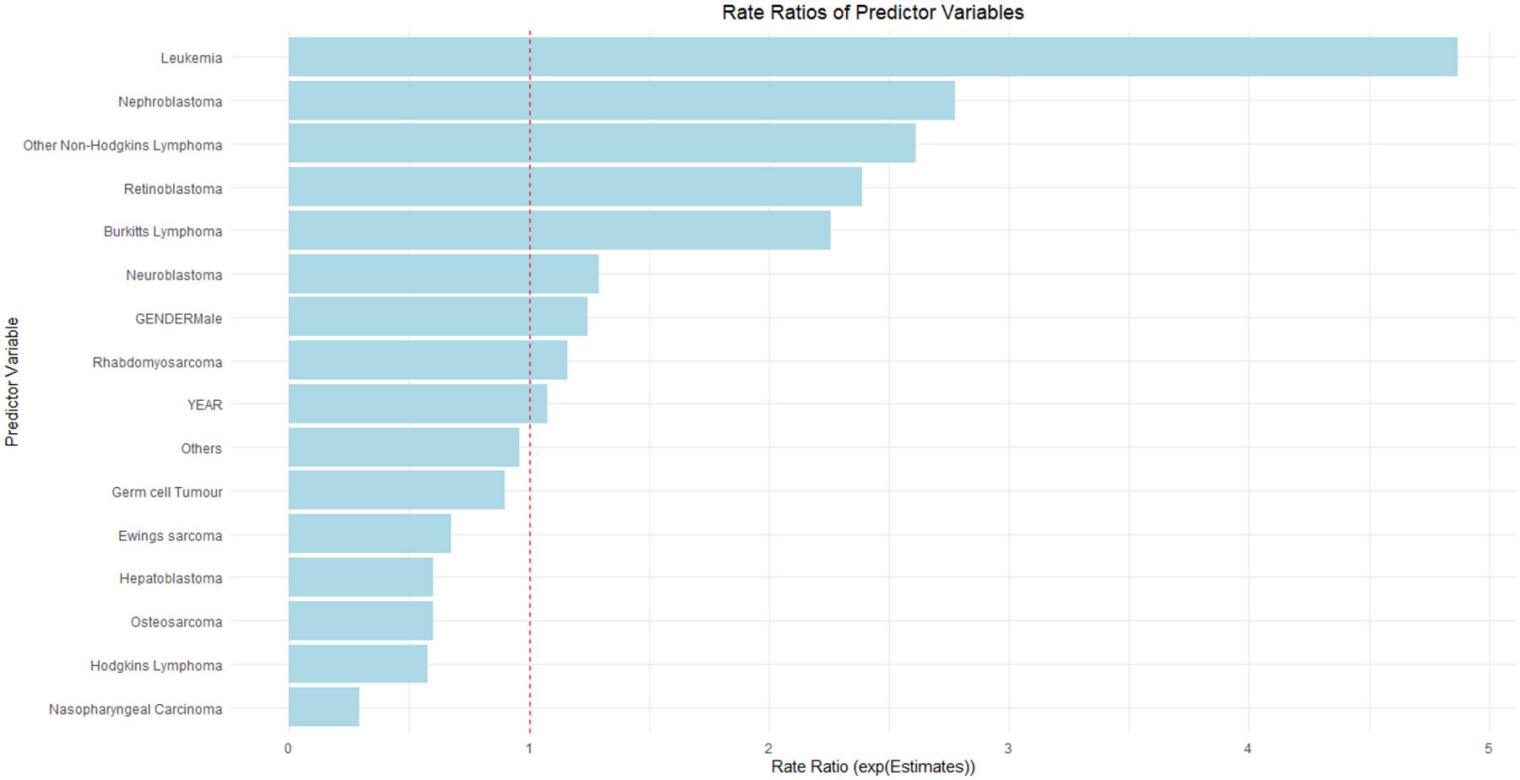
Rate ratios of predictor variables relative to the mean (red line).

The result of the Poisson regression indicates a positive upward trend over time (IRR: 1.08, *p*: < 0.001, CI: 1.02, 1.14), suggesting an increasing trend in pediatric cancer cases from 2018 to 2022. Compared to the reference category, the incidence of Leukemia (IRR: 4.87, *p*: < 0.001, CI: 3.31, 7.17), Burkitts Lymphoma (IRR: 2.26, *p*: < 0.001, CI: 1.48, 3.45), Nephroblastoma (IRR: 1.29, *p*: < 0.001, CI: 0.80, 2.06), Other Non‐Hodgkins Lymphoma (IRR: 2.61, *p*: < 0.001, CI: 1.72, 3.95), and Retinoblastoma (IRR: 2.39, *p*: < 0.001, CI: 1.57, 3.63) all have highly significant positive estimates and thus are more common compared to the other cancer types such as Nasopharyngeal Carcinoma (IRR: 0.29, *p*: 0.091, CI: 0.06, 1.22) and Hodgkins Lymphoma (IRR: 0.58, *p*: 0.08, CI: 0.31, 1.07). Males showed a higher incidence of cancer cases compared to females, with IRR estimates of 1.24 at *p*: < 0.01, CI: 1.06, 1.45, suggesting that males have a 24% higher incidence rate compared to females, as shown in Figure 3.

**Figure 4:**
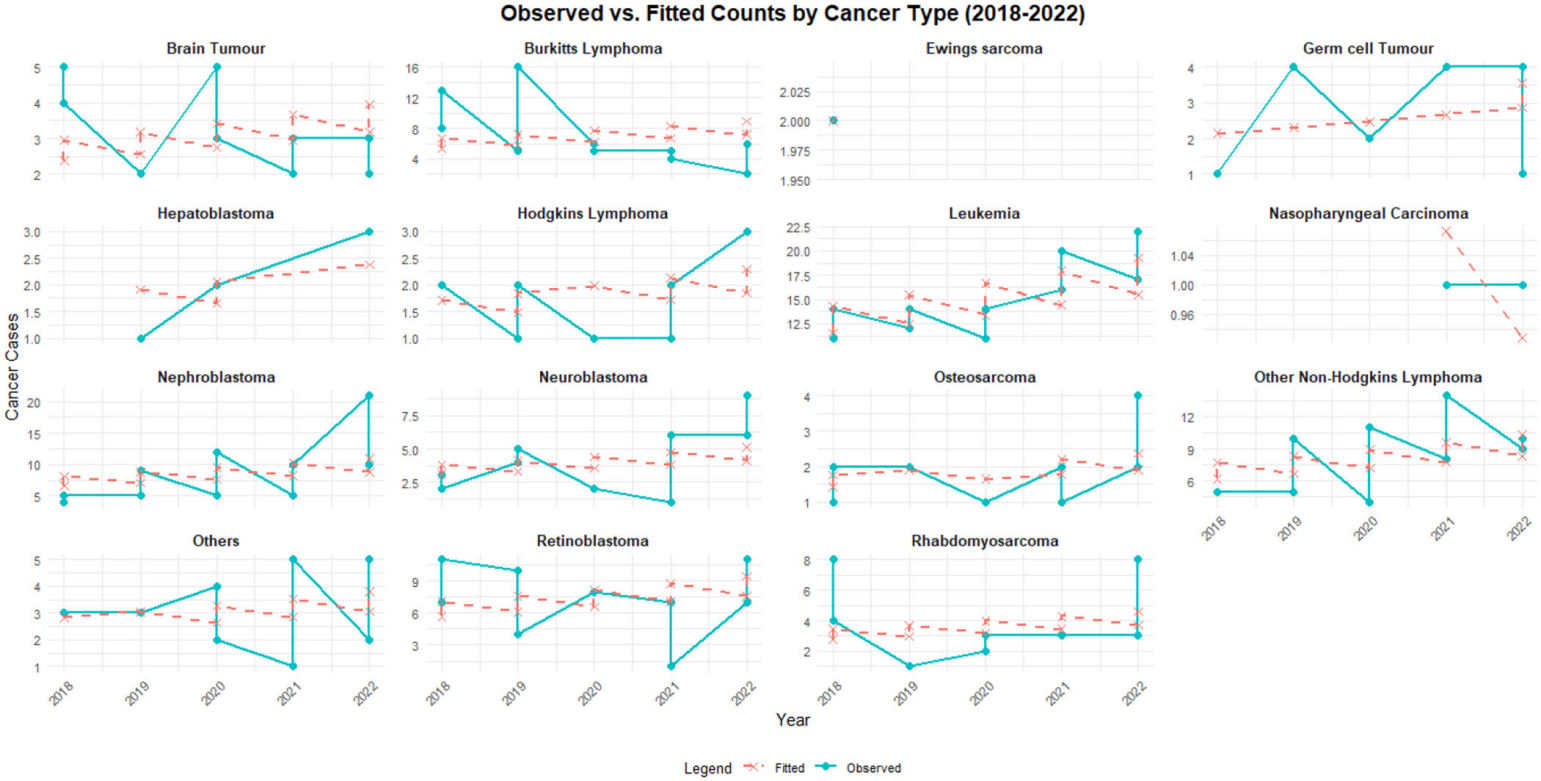
Observed versus fitted cancer cases by type (2018–2022).

A further comparison of observed and fitted values reveals a good model fit that adequately captures the temporal trend in cancer incidence across cancer types and gender categories, as shown in Figure 4. The test for overdispersion was reported at (∅ = 1.01, *p* = 0.477), confirming the appropriateness of the Poisson model.

### 3.1 Point Process Model of Pediatric Cancer

**Figure 5:**
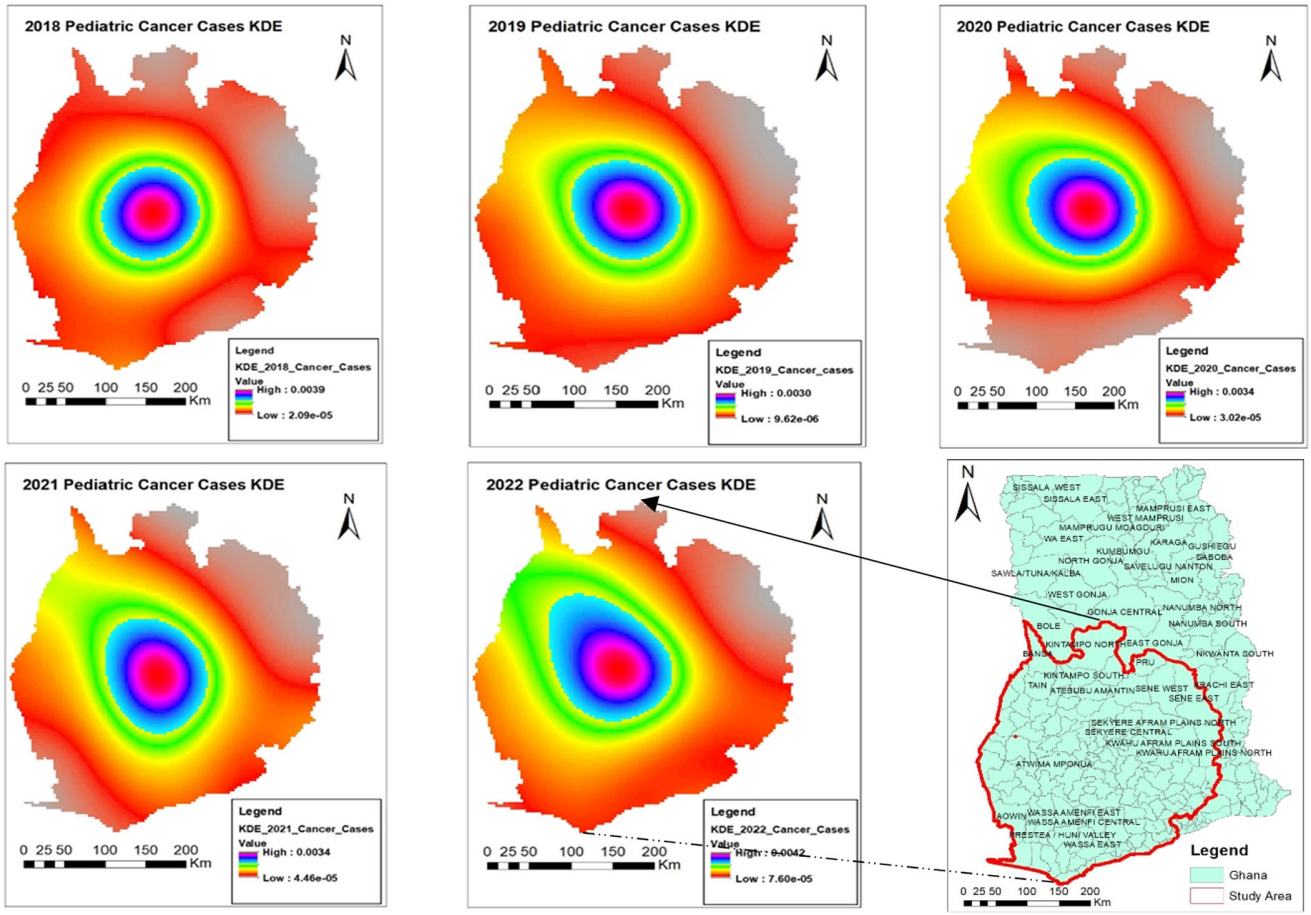
Temporal density estimates of pediatric cancer in Southern Ghana.

KDE plots from 2018 to 2022 consistently reveal a high density of reported cases in the central part of the study area, highlighting the Ashanti region as a significant hotspot for pediatric cancer incidences. Comparing the 2018 KDE plot to those of subsequent years reveals a noticeable directional diffusion of pediatric cancer cases toward the northwest, as shown in Figure 5. This shift becomes increasingly pronounced from 2019 to 2022, indicating rising high‐density values.

### 3.2 Cluster Analysis of Pediatric Cancer

**Figure 6:**
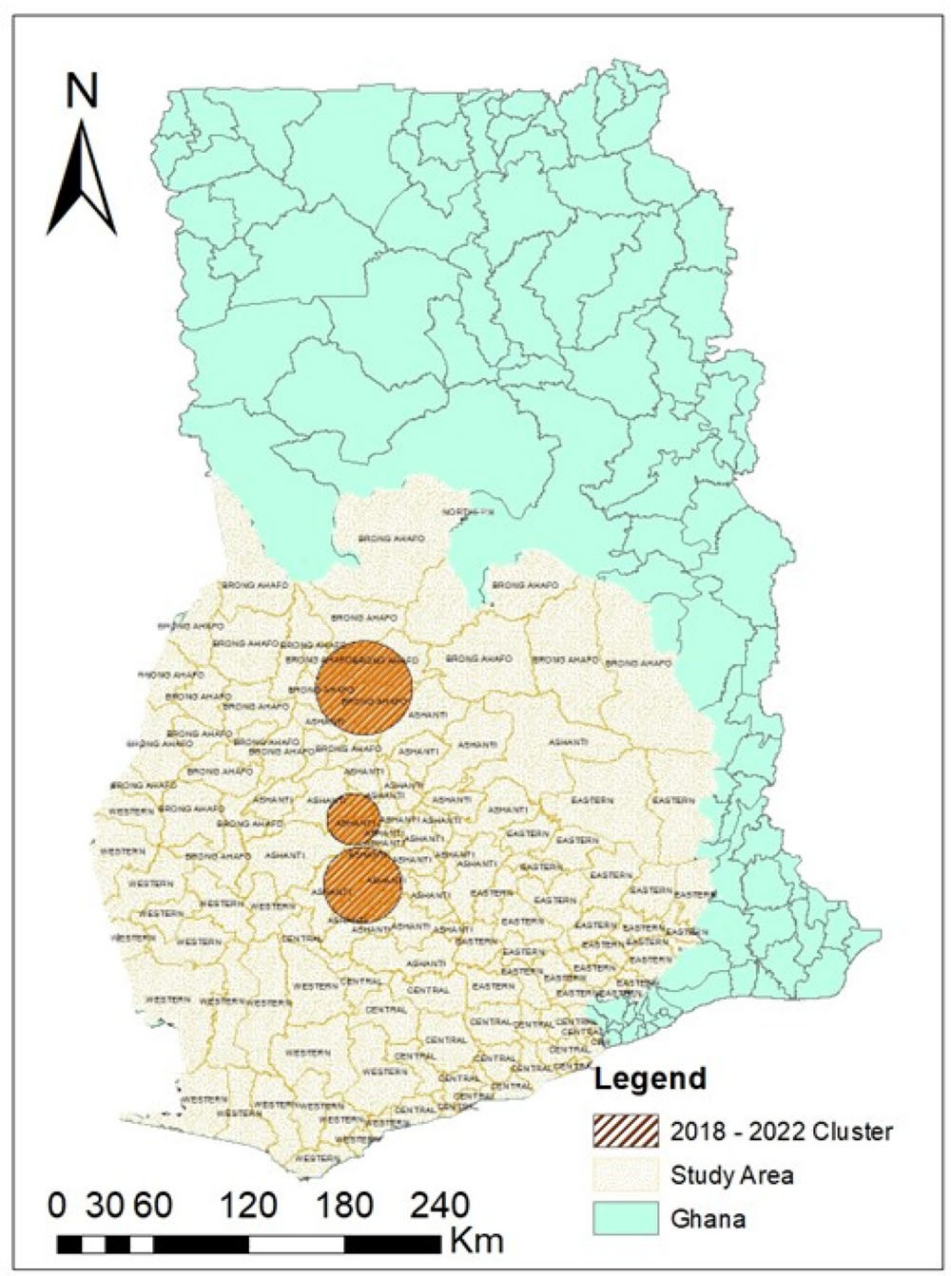
Clusters from 5‐year pediatric cancer cases (2018–2022).

The proximity and spatial relationships between individual points were studied to identify statistically significant clusters or deviations from random spatial patterns in the study area. Considering the 5‐year study period together, three (3) significant clusters were identified in these illegal mining‐endemic districts, including Techiman North, Nkoranza, Atwima‐ Nwabiagya, Kwanwoma, Amansie‐West and Central, and Bekwai Municipal, as seen in Figure 6, with expected cases ranging from 1.06 to 4.96, and relative risk from 1.65 to 2.17 at *p* < 0.01.

**Figure 7:**
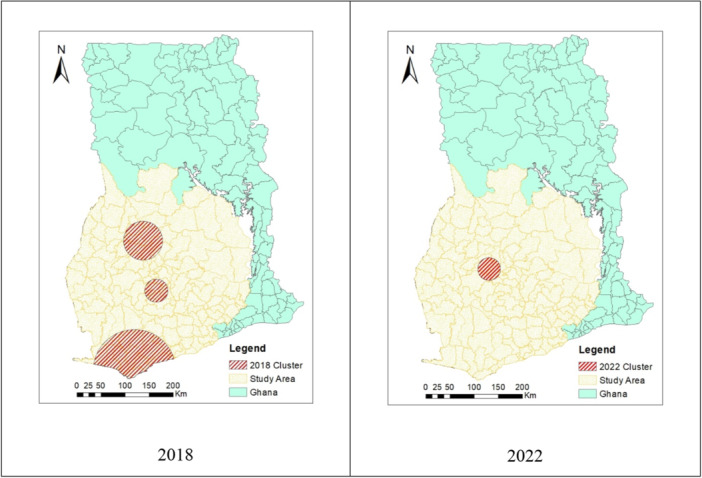
Map of 2018 and 2022 pediatric cancer clusters.

Further, we considered beginning and end timestamps and cancer cases for 2018 and 2022, examining them independently for clustering, as shown in Figure 7. In 2018, three (3) major clusters (constituting Amansie‐West, Central, Bekwai Municipal, Atwima‐Nwabiagya, and Asafo Ano South districts) were identified. However, these were not significant, with expected cases ranging from 0.34 to 4.02 and the relative risk of 2.67–8.33 at *p* > 0.1. In 2022, a single cluster was found to cover Afigya Kwabre and Sekyere, Atwima Kwanwoma and Nwabiagya, Offinso Municipal, Kwabre, Ahafo Ano South, and KMA districts in the Ashanti Region. Again, this was not significant with expected cases of 3.10 and a relative risk of 1.56 at *p* > 0.1.

## 4 Discussion

Data from KATH reveals an alarming increase in pediatric cancer cases in Ghana. This could be due to improved diagnostics and awareness about the disease. In addition, predictions by Wang et al. [23] have shown a slight increase in incidence from 2020. In our study, we found an overall increase in the incidences of pediatric cancers among males compared to females between the ages of 0–17 years. This is comparable to studies by Marcotte et al. [8]; Williams et al. [9], who also found an increase in the incidence of pediatric cancers in males. These observed gender‐related variations underscore the importance of gender‐sensitive cancer surveillance and research, particularly in populations with unique demographic and health profiles. Leukemia was the most dominant and persistent cancer type reported in the study area and did not discriminate among gender types [24].

Each year, the KDE plots for pediatric cancer highlighted the Ashanti region as a significant hotspot for pediatric cancer incidences, especially in the Bekwai and Atwima Nwabiagya Districts. While KDE is sensitive to bandwidth selection and assumes spatial continuity, its adaptability makes it a valuable tool for exploratory spatial analysis [21]. These hotspots could be attributed to several factors, including proximity to the KATH, increased awareness of cancers amongst the populace, and subsequent reporting to the hospital. This may be a result of the improved diagnostic facilities at KATH over the years. This persistent pattern over multiple years underscores the need for focused public health interventions and further investigation into potential environmental (man‐made or natural) or socioeconomic factors contributing to this concentration. Coincidentally, studies have shown that these districts are hotspots for illegal mining activities in Ghana, most of which do not follow sustainable environmental principles [25, 26]. The environmental perturbations, including heavy metal exposure, associated with these unregulated mining practices represent a potential, yet understudied, risk factor for pediatric malignancies. Future epidemiological and toxicological studies are crucial to establish a potential causal relationship.

Additionally, socioeconomic factors, which include financial constraints, limited health literacy, and geographical barriers, influence healthcare access and reporting patterns, potentially leading to underreporting in rural areas and clustering around healthcare facilities [27, 28]. Identifying and addressing these localized risk factors is essential for effective early diagnosis and treatment strategies for pediatric cancer in the region.

The results of the SaTScan analysis for the spatial patterns of cancer data from 2018 to 2022 indicate significant spatial clustering. The test for spatial dependency for the start and end years, i.e., 2018 and 2022, also revealed major clusters. However, these were not statistically significant, which could be attributed to the limited sample sizes in the individual years, limiting the ability to detect clusters [29, 30]. These clusters show that pediatric cancer is not equally likely to occur at any location within the study area. The consistency strengthens the evidence for nonrandomness and suggests that underlying spatial factors may contribute to the spatial clustering of pediatric cancer cases throughout these years. These factors could include environmental exposures to carcinogens, genetic predisposition, socioeconomic disparities, and changes in lifestyle and behavior over time [31]. In Southern Ghana, particularly the Ashanti Region, where mining is prevalent, potential environmental risk factors such as chemical exposures and heavy metals toxicity may play a role, alongside the complex interactions between genes and the environment in the etiology of childhood cancers [32]. Elsewhere, environmental risk factors have been implicated in pediatric cancers, with radiation and chemical exposures, and certain parental exposures suggested [33]. Most childhood cancers are believed to result from complex interactions between genes and the environment after conception, highlighting the importance of investigating potential environmental influences on pediatric cancer incidence [10].

A key limitation of this study is that it relies on hospital‐reported cancer cases, which may not capture undiagnosed or untreated cases, particularly in rural areas where access to healthcare is limited. However, focusing on documented cases with confirmed diagnoses provides the most reliable spatial data for analysis. Additionally, the observed spatial clustering of pediatric cancer cases may be influenced by environmental exposures, genetic predisposition, and socioeconomic disparities, which were not captured in this study. Despite these limitations, the findings provide valuable insights into the spatiotemporal patterns of cancer cases and highlight potential areas for targeted healthcare interventions in Ghana.

## 5 Conclusions

In the present study, pediatric cancer incidences from 2018 to 2022 in KATH reveal significant spatial clustering, particularly in the Ashanti Region and increasingly towards the northwest. A higher incidence among males and increasing leukemia cases indicate an urgent need for public health intervention in these areas. Hotspots at specific locations identified in the study area underscore the need for targeted environmental research and strategies to mitigate these risks and improve outcomes in affected populations.

## Author Contributions

**Efiba Vidda Senkyire Kwarteng:** conceptualization, writing – review and editing, writing – original draft, methodology, formal analysis. **Amma Aboagyewa Larbi:** conceptualization, writing – original draft, writing – review and editing. **Nanatte Veronica Anderson:** writing – review and editing, formal analysis. **Sheriff Wahab:** methodology, formal analysis. **Yaa Gyamfua Oppong‐Mensah:** writing – review and editing. **Samuel Ato Andam‐Akorful:** conceptualization, writing – original draft, writing – review and editing, methodology, formal analysis, supervision. **Alexander Kwarteng:** writing – review and editing. **Lawrence Osei‐Tutu:** writing – review and editing, writing – original draft. **Gladys Acquah:** writing – review and editing. **Comfort Asoogo:** writing – review and editing. **Bernice Eklu:** writing – review and editing. **Paul Obeng:** writing – review and editing. **Barnabas Manlokiya:** writing – review and editing. **Ben Gyan:** writing – review and editing. **Vivian Paintsil:** conceptualization, writing – original draft, writing – review and editing, methodology, supervision.

## Ethics Statement

The authors have nothing to report.

## Conflicts of Interest

The authors declare no conflicts of interest.

## Declarations

All authors have read and approved the final version of the manuscript. Efiba Vidda Senkyire Kwarteng has full access to all the data in this study and takes complete responsibility for the integrity of the data and the accuracy of the data analysis.

## Transparency Statement

The lead author Amma Aboagyewa Larbi affirms that this manuscript is an honest, accurate, and transparent account of the study being reported; that no important aspects of the study have been omitted; and that any discrepancies from the study as planned (and, if relevant, registered) have been explained.

## Data Availability

The data that support the findings of this study are available from the corresponding author upon reasonable request. The original contributions presented in the study are included in the manuscript. Further inquiry can be directed at the corresponding author.
